# Tai Chi as a public health intervention: effects on stress regulation, attention, and psychological resilience in adults

**DOI:** 10.3389/fpubh.2026.1791759

**Published:** 2026-05-04

**Authors:** Nan Wang, Zi Yang

**Affiliations:** School of Kinesiology and Physical Education, Zhengzhou University, Henan, China

**Keywords:** attention, mind-body therapies, resilience, stress, Tai Chi

## Abstract

**Background:**

Chronic psychological stress, impaired attentional functioning, and reduced psychological resilience represent major public health challenges among adults, contributing to adverse mental and physical health outcomes. Mind–body interventions have gained increasing attention as scalable, non-pharmacological strategies to address these interconnected domains. Tai Chi, a traditional Chinese mind–body practice characterized by slow movements, controlled breathing, and mindful attention, is particularly suitable for population-level implementation due to its low cost, accessibility, and adaptability.

**Methods:**

This randomized controlled trial evaluated the effects of an 8-week structured Tai Chi intervention on stress regulation, attention, and psychological resilience in adults aged 30–55 years. Sixty-eight participants were randomly assigned to either a Tai Chi intervention group (TCG, *n* = 34) or a control group (CG, *n* = 34). The TCG participated in supervised Tai Chi sessions three times per week (60 min/session), while the CG continued routine daily activities. Outcome measures included the Perceived Stress Scale (PSS-10) for stress regulation, the Attention Network Test (ANT) for attentional performance, and the Connor–Davidson Resilience Scale (CD-RISC-25) for psychological resilience. Assessments were conducted at baseline and post-intervention. Data were analyzed using repeated-measures ANOVA with effect sizes reported as partial eta squared (η^2^p).

**Results:**

All variables demonstrated normal distribution at baseline. Significant Group × Time interaction effects were observed for stress regulation (η^2^p = 0.32), attention (η^2^p = 0.16), and psychological resilience (η^2^p = 0.41), indicating greater improvements in the Tai Chi group compared with controls. *Post hoc* analyses revealed that the TCG exhibited a significant reduction in perceived stress, faster attentional reaction times, and a substantial increase in psychological resilience following the intervention (*p*<*0.01*), whereas no significant changes were observed in the control group.

**Discussion:**

The findings provide robust evidence that Tai Chi is an effective and potentially scalable mind-body intervention for improving stress regulation, attentional performance, and psychological resilience in adults. The moderate-to-large effect sizes observed across outcomes highlight Tai Chi's potential as a holistic mind–body strategy for enhancing psychological wellbeing.

## 1 Introduction

Chronic psychological stress has become a pervasive public health concern among adults, driven by increasing occupational demands, sedentary lifestyles, and psychosocial pressures. Prolonged stress exposure is associated with dysregulation of the hypothalamic-pituitary-adrenal (HPA) axis, impaired autonomic balance, inflammation, and elevated risk of mental and physical disorders, including depression, cardiovascular disease, and cognitive decline (1, 2). In parallel, deficits in attentional control and reduced psychological resilience further exacerbate vulnerability to stress-related pathology, limiting individuals' capacity to adapt effectively to environmental demands (3, 4). As a result, scalable and preventive interventions that enhance stress regulation, attention, and resilience are increasingly prioritized within public health frameworks.

Mind-body interventions represent a promising non-pharmacological approach to addressing these interconnected outcomes. Practices such as Tai Chi, yoga, and meditation integrate physical movement with attentional focus and controlled breathing, targeting both physiological and psychological processes underlying health and wellbeing (5, 6). Among these, Tai Chi has received growing attention due to its low-impact nature, adaptability across age groups, and minimal resource requirements, making it particularly suitable for large-scale community and workplace implementation.

Tai Chi is a traditional Chinese mind-body exercise characterized by slow, flowing movements, postural alignment, diaphragmatic breathing, and sustained mindful attention. From a mechanistic perspective, Tai Chi has been shown to influence stress regulation by enhancing parasympathetic activity, reducing sympathetic arousal, and attenuating cortisol responses, thereby promoting psychophysiological homeostasis (7, 8). Meta-analytic evidence indicates that Tai Chi interventions produce significant reductions in perceived stress, anxiety, and depressive symptoms across diverse adult populations, including healthy individuals and those with chronic conditions (9, 10).

Beyond stress reduction, Tai Chi may positively influence attentional functioning and cognitive performance. The continuous requirement to coordinate movement, breath, and mental focus during Tai Chi practice engages attentional networks associated with alerting, orienting, and executive control (11). Empirical studies have reported improvements in attention, working memory, processing speed, and executive functioning following Tai Chi training, particularly in middle-aged and older adults (7, 12). Such findings suggest that Tai Chi may serve as a cognitively enriching physical activity with relevance for cognitive health promotion.

Psychological resilience, defined as the capacity to adapt successfully to stress and adversity, represents another key outcome relevant to public health (4). Mind-body practices have been shown to enhance resilience by fostering emotional regulation, self-efficacy, and adaptive coping strategies (13, 14). Emerging evidence indicates that Tai Chi participation is associated with greater resilience, improved mood stability, and enhanced perceived coping ability, likely mediated through improvements in mindfulness and stress appraisal processes (10, 15).

Despite growing evidence supporting the psychological benefits of Tai Chi, relatively few randomized controlled trials have simultaneously examined its effects on stress regulation, objective attentional performance, and psychological resilience within a single integrated framework, particularly among working-age adults. Most previous studies have focused on isolated outcomes such as stress reduction or cognitive performance separately. Therefore, the present study aimed to evaluate the effectiveness of an 8-week structured Tai Chi intervention on stress regulation, attention, and psychological resilience using a randomized controlled design. By simultaneously examining these multidimensional psychological outcomes and conceptualizing Tai Chi as a potentially scalable mind-body intervention for community-dwelling adults, the present study extends existing literature and provides a more comprehensive understanding of Tai Chi's role in promoting psychological wellbeing and cognitive functioning.

## 2 Materials and methods

### 2.1 Experimental design

**Figure 1:**
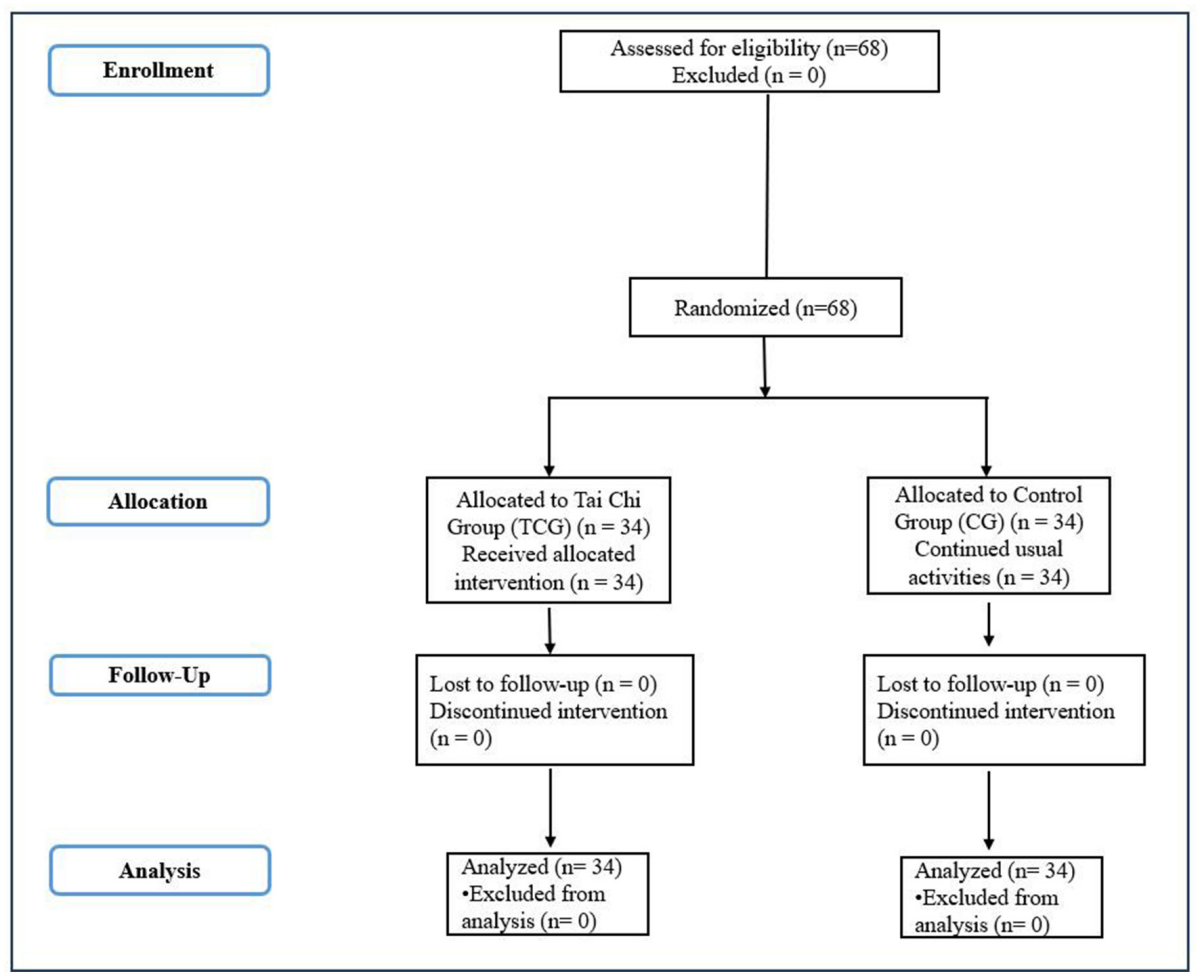
CONSORT flow diagram of participant recruitment, randomization, allocation, follow-up, and analysis.

This study adopted a randomized controlled trial (RCT) design to evaluate the effectiveness of Tai Chi as a potentially scalable mind-body intervention on stress regulation, attention, and psychological resilience among adults. Participants were recruited using a purposive voluntary sampling technique, and sample size was determined *a priori* using G^*^Power software (version 3.1) for a repeated-measures ANOVA (within-between interaction), assuming a medium effect size (f = 0.25), α = 0.05, statistical power (1–β) = 0.80, two groups, and two measurement points, which indicated a minimum required sample of 54 participants; to account for potential attrition, additional participants were recruited, resulting in a total sample of *N* = 68. Participants were then randomly allocated using a computer-generated random sequence into either a Tai Chi Intervention Group (TCG, *n* = 34) or a Control Group (CG, *n* = 34). The flow of participants through the stages of recruitment, randomization, allocation, follow-up, and analysis is illustrated in Figure 1. The Tai Chi group participated in an 8-week structured Tai Chi training program, while the control group continued with routine daily activities without any structured intervention. Outcome measures were assessed at baseline (pre-intervention) and post-intervention, and randomization ensured baseline equivalence between groups, minimizing allocation bias, with demographic characteristics confirmed to be comparable across groups. The study protocol, including participant recruitment procedures, intervention design, and outcome measures, was established prior to the initiation of the study and approved by the Institutional Ethics Committee. Although the study followed a randomized controlled trial design, the trial was not prospectively registered in a clinical trial registry. Future studies should consider prospective trial registration to enhance transparency and alignment with international reporting standards.

This figure illustrates the flow of participants through the randomized controlled trial. A total of 68 adults were assessed for eligibility and randomly allocated to either the Tai Chi intervention group (TCG, *n* = 34) or the control group (CG, *n* = 34). Participants in the TCG received an 8-week structured Tai Chi training program, while participants in the CG continued with routine daily activities. No participants were lost to follow-up, and all randomized participants were included in the final analysis.

### 2.2 Participants of the study

A total of 68 adults (male and female), aged 30–55 years, participated in this study. Participants were recruited from community centers, workplaces, and residential areas in Zhengzhou University, China through voluntary participation and were screened prior to enrollment to ensure suitability for a low- to moderate-intensity mind-body intervention. Eligible participants had no prior formal experience with Tai Chi or other structured mind-body practices and were generally healthy at the time of recruitment. Inclusion criteria comprised adults within the specified age range who were physically capable of performing gentle movements and who provided written informed consent. Exclusion criteria included a history of diagnosed psychiatric, neurological, or severe musculoskeletal disorders, current use of psychotropic or stress-modulating medications, regular engagement in yoga, meditation, or similar interventions during the study period, or any medical condition that could compromise safe participation. Following eligibility screening, participants were randomly allocated to either the Tai Chi Intervention Group (TCG, *n* = 34) or the Control Group (CG, *n* = 34), with Baseline demographic characteristics, including age, height, weight, body mass index, and sex distribution, were comparable between the Tai Chi intervention and control groups, with no statistically significant differences observed Table 1.

**Table 1:** Baseline demographic characteristics of participants.

Variable	Tai Chi intervention group (TCG, *n* = 34)	Control group (CG, *n* = 34)	*p*-value
Age (years)	42.6 ± 6.8	43.1 ± 7.2	*0.74*
Height (cm)	168.4 ± 7.9	169.1 ± 8.2	*0.68*
Weight (kg)	69.8 ± 9.6	70.5 ± 10.1	*0.76*
Body mass index (kg/m^2^)	24.6 ± 2.8	24.8 ± 3.0	*0.81*
Sex (male/female)^†^	18/16	17/17	*0.82*

Values are presented as mean ± standard deviation unless otherwise indicated. ^†^Sex distribution presented as frequency. *p* < 0.05 denotes statistical significance.

### 2.3 Intervention protocol

Participants allocated to the Tai Chi Intervention Group (TCG) underwent a structured 8-week Tai Chi training program designed to promote stress regulation, attentional control, and psychological resilience through mindful movement and controlled breathing. The intervention followed the 24-form simplified Yang-style Tai Chi, which is widely used in clinical and public health research due to its accessibility, moderate intensity, and standardized sequence of movements. This protocol has been frequently adopted in previous Tai Chi intervention studies examining psychological and physiological outcomes. All sessions were conducted in a group format and supervised by a certified Tai Chi instructor with more than 5 years of teaching experience in Tai Chi training to ensure correct technique, participant safety, and adherence to the intervention protocol. A detailed description of the Tai Chi intervention protocol, including session structure, frequency, and duration, is presented in Table 2.

**Table 2:** Tai Chi intervention protocol and control condition description.

Component	Tai Chi intervention group (TCG)	Control group (CG)
Study duration	8 weeks	8 weeks
Session frequency	3 sessions per week	No structured sessions
Session duration	60 min per session	Not applicable
Intervention type	Structured Tai Chi program	Routine daily activities
Warm-up phase	10 min (joint mobility and gentle stretching)	Not applicable
Core activity	40 min of simplified Tai Chi practice emphasizing slow movements, postural alignment, controlled breathing, and mindful attention	Not applicable
Cool-down phase	10 min (relaxation and breathing exercises)	Not applicable
Supervision	Sessions supervised by a qualified Tai Chi instructor	No supervision
Exercise intensity	Low to moderate	Not applicable
Additional instructions	Participants encouraged to attend all sessions and maintain usual lifestyle habits	Participants instructed not to engage in any new exercise, Tai Chi, meditation, or mind–body practices

The Tai Chi training program incorporated gradual progression throughout the 8-week intervention period. During the first 2 weeks, participants were introduced to fundamental Tai Chi principles including posture alignment, breathing coordination, and basic movement patterns. Weeks 3–5 focused on learning additional movement sequences within the 24-form routine while emphasizing coordination between movement and breathing. During weeks 6–8, participants practiced longer movement sequences and complete form routines with improved movement fluidity, balance control, and attentional focus. Although the overall physical intensity remained low-to-moderate, progression was achieved through increasing movement complexity, improved coordination, and enhanced mindfulness engagement during practice. Although the overall physical intensity remained low-to-moderate, progression was achieved through increasing movement complexity, improved coordination, and enhanced mindfulness engagement during practice.

Participant adherence to the intervention program was monitored through attendance records maintained during each training session. Participants were required to attend at least 80% of the scheduled sessions to be included in the final analysis. All participants met this attendance criterion, and therefore no participants were excluded from the analysis due to insufficient participation.

Participants in the Control Group (CG) did not receive any structured physical or mind–body intervention during the study period and were instructed to continue with their usual daily activities. They were advised not to initiate any new exercise, Tai Chi, meditation, or relaxation practices throughout the intervention duration. Adherence to the intervention was monitored through attendance records, and participants attending fewer than the predefined minimum number of sessions were excluded from the final analysis.

### 2.4 Outcome measures

The primary outcome measures of the study included stress regulation, attention, and psychological resilience. All outcomes were assessed at baseline (pre-intervention) and after completion of the 8-week intervention (post-intervention) using standardized, validated, and widely accepted psychological assessment tools. The selected instruments have demonstrated strong psychometric properties and have been extensively used in adult and public health research, including studies conducted within Chinese populations.

#### 2.4.1 Stress regulation

Stress regulation was assessed using the Perceived Stress Scale (PSS-10) (16). The PSS-10 is a widely used self-report instrument designed to measure the degree to which individuals perceive their life situations as stressful over the past month (17). The scale consists of 10 items rated on a 5-point Likert scale ranging from 0 (never) to 4 (very often), with higher scores indicating greater perceived stress and poorer stress regulation. The PSS-10 has demonstrated strong internal consistency (Cronbach's α = 0.78–0.91), test–retest reliability, and cross-cultural validity (18). Validated Chinese versions of the PSS-10 have shown excellent psychometric properties in mainland Chinese populations (19, 20), making it suitable for public health intervention studies examining stress-related outcomes.

#### 2.4.2 Attention

Attentional functioning was measured using the Attention Network Test (ANT) (21), a computerized cognitive task that provides objective indices of attentional performance. The ANT evaluates three core components of attention, alerting, orienting, and executive control, by recording reaction time and accuracy responses to visual stimuli presented under various cueing and flanker conditions (22). Participants completed the task in a controlled environment following standardized instructions. Faster reaction times and higher accuracy scores reflect better attentional control and cognitive efficiency (11). The ANT is considered an advanced and sensitive measure of attention and has been extensively applied in cognitive and neuropsychological research, demonstrating robust psychometric properties and cross-cultural reliability (23, 24).

#### 2.4.3 Psychological resilience

Psychological resilience was assessed using the Connor-Davidson Resilience Scale (CD-RISC-25) (25). This scale consists of 25 items rated on a 5-point Likert scale (0 = not true at all to 4 = true nearly all the time), measuring adaptability, emotional strength, persistence, and coping capacity in response to stress and adversity. Higher scores indicate greater psychological resilience (range: 0–100). The CD-RISC has demonstrated excellent reliability (Cronbach's α = 0.89), construct validity, and sensitivity to treatment effects across diverse populations (26, 27). Validated Chinese versions of the CD-RISC-25 have shown strong psychometric properties in adult populations, with documented internal consistency (α = 0.91) and test-retest reliability (28, 29), supporting its use in Chinese community samples.

All assessments were administered by trained investigators who were blinded to group allocation to minimize assessment bias. Participants completed the self-report questionnaires (PSS-10 and CD-RISC-25) in a quiet environment, while computerized attention testing (ANT) was conducted under standardized laboratory conditions to ensure consistency and data reliability.

### 2.5 Data collection procedure

Data collection was conducted in a standardized and controlled manner at two time points: baseline (pre-intervention) and post-intervention (after 8 weeks). All participants completed the psychological questionnaires assessing stress regulation and psychological resilience in a quiet environment under researcher supervision, while attentional performance was evaluated using a computerized task administered in a controlled setting following standardized instructions. Trained assessors who were blinded to group allocation conducted all assessments to minimize measurement bias. Participants were instructed to avoid strenuous physical activity and caffeine intake for at least 12 h prior to testing. Attendance records were maintained throughout the intervention period to monitor adherence, and only participants meeting the predefined attendance criteria were included in the final analysis.

### 2.6 Ethical approval and informed consent

The study protocol was reviewed and approved by Professorial Committee of the School of Physical Education and Sports, Zhengzhou University, China (Approval No. ZZUIRB2026-01), and was conducted in accordance with the ethical principles outlined in the Declaration of Helsinki. Prior to participation, all individuals received detailed information regarding the study objectives, procedures, potential risks, and benefits. Written informed consent was obtained from all participants before the commencement of data collection. Participation was entirely voluntary, confidentiality was ensured through anonymized data coding, and participants were informed of their right to withdraw from the study at any stage without any consequences.

### 2.7 Statistical analysis

Statistical analysis was performed using SPSS software (version 23). Data were screened for normality using the Shapiro-Wilk test (See Table 3), and descriptive statistics were computed as mean ± standard(pre-post) and between-group differences for all dependent variables. Effect sizes were calculated using (pre-post) and between-group differences for all dependent variables. Effect sizes were calculated using partial eta squared (η^2^p) to determine the magnitude of intervention effects. The level of statistical significance was set at *p* < 0.05 for all analyses. Bonferroni-adjusted *post hoc* comparisons were conducted to control for potential Type I error arising from multiple outcome testing.

**Table 3:** Shapiro-wilk normality test for pre-intervention scores.

Variable	Group	W statistic	*p*-value
Stress regulation	TCG	0.973	0.559
	CG	0.962	0.279
Attention	TCG	0.959	0.227
	CG	0.959	0.229
Psychological resilience	TCG	0.989	0.977
	CG	0.979	0.735

## 3 Results

The Shapiro-Wilk test was conducted to examine the normality of pre-intervention data for all dependent variables in both the Tai Chi intervention group (TCG) and the control group (CG). All *p*-values exceeded the 0.05 significance level, indicating that stress regulation, attention, and psychological resilience were normally distributed at baseline. These results confirm that the assumption of normality was satisfied, supporting the use of parametric statistical analyses.

Table 4 presents the descriptive statistics for stress regulation, attention, and psychological resilience in the Tai Chi intervention group (TCG) and the control group (CG) at pre- and post-intervention assessments. At baseline, mean scores were comparable between groups across all variables. Following the intervention, the TCG demonstrated a noticeable reduction in stress regulation scores, accompanied by improvements in attentional performance and psychological resilience. In contrast, the CG showed minimal changes from pre- to post-test across all outcome measures. These descriptive trends suggest favorable changes in psychological outcomes associated with the Tai Chi intervention.

**Table 4:** Descriptive statistics (Mean ± SD) for pre- and post-intervention scores in Tai Chi and control groups.

Variable	Group	Pre-test	Post-test
Stress regulation	TCG	21.36 ± 3.72	16.64 ± 3.69
	CG	21.57 ± 3.60	21.73 ± 4.22
Attention	TCG	554.74 ± 55.04	506.98 ± 66.32
	CG	549.35 ± 52.84	532.78 ± 69.49
Psychological resilience	TCG	60.74 ± 6.59	70.24 ± 6.00
	CG	62.48 ± 6.20	62.03 ± 8.97

Table 5 presents the results of the repeated-measures ANOVA examining the effects of time, group, and their interaction on stress regulation, attention, and psychological resilience. Significant main effects of time were observed for all variables (*p* < 0.001), indicating overall changes from pre- to post-intervention. Significant group effects were also found, demonstrating differences between the Tai Chi intervention group and the control group. Importantly, significant Group × Time interaction effects were observed for all outcome measures, with moderate to large effect sizes (η^2^p = 0.16–0.41), indicating that the Tai Chi intervention produced significantly greater improvements in stress regulation, attentional performance, and psychological resilience compared with the control condition.

**Table 5:** Repeated-measures ANOVA results for time, group, and group × time effects on psychological variables.

Variable	Effect	F (1,66)	*p*-value	η^2^p
Stress regulation	Time	22.29	* < 0.001*	0.25
	Group	9.84	*0.003*	0.13
Group × time	31.12	* < 0.001*	0.32
Attention	Time	14.67	* < 0.001*	0.18
	Group	4.92	*0.030*	0.07
Group × time	12.88	*0.001*	0.16
Psychological resilience	Time	40.86	* < 0.001*	0.38
	Group	10.73	*0.002*	0.14
Group × time	45.19	* < 0.001*	0.41

*p* < 0.05 denotes statistical significance.

Table 6 presents the Bonferroni-adjusted *post hoc* comparisons examining within-group pre-post changes for psychological variables. The Tai Chi intervention group (TCG) demonstrated statistically significant improvements across all outcomes, including a significant reduction in stress regulation scores and attention reaction time, along with a significant increase in psychological resilience (*p*<*0.001*). In contrast, the control group (CG) showed no significant changes across any variables. These findings further confirm that the observed improvements were specific to the Tai Chi intervention and not attributable to natural variation over time.

**Table 6:** Bonferroni-adjusted *post hoc* within-group comparisons (pre-post changes).

Variable	Group	Mean difference (post—pre)	Std. error	*p*-value (Bonferroni)
Stress regulation	TCG	−4.72	0.99	< 0.001^*^
	CG	0.16	1.02	1.000
Attention	TCG	−47.76	13.45	0.001^*^
	CG	−16.57	13.80	0.262
Psychological resilience	TCG	9.50	1.49	< 0.001^*^
	CG	−0.45	1.54	0.817

^*^ indicates statistically significant differences after Bonferroni adjustment (*p* < 0.05).

## 4 Discussion

The present study investigated the effects of Tai Chi as a potentially scalable mind-body intervention on stress regulation, attention, and psychological resilience in adults. The findings provide robust evidence supporting the efficacy of Tai Chi in improving multiple dimensions of psychological wellbeing. Significant Group × Time interactions were observed across all outcome measures, with moderate to large effect sizes (η^2^p = 0.16–0.41), indicating that participants in the Tai Chi intervention group demonstrated substantially greater improvements compared to the control group. These results align with and extend the existing literature on mind-body interventions, while offering important implications for public health practice.

The Tai Chi intervention group exhibited a significant reduction in stress regulation scores (M = −4.72, *p* < 0.001), whereas the control group showed no meaningful change. This finding is consistent with previous research demonstrating the stress-reducing effects of Tai Chi practice. A systematic review and meta-analysis by Wang et al. (10) reported that Tai Chi interventions significantly reduced stress and anxiety symptoms across diverse populations, with effect sizes comparable to those observed in the present study. Similarly, a randomized controlled trial by Motivala et al. (30) found that 16 weeks of Tai Chi practice led to significant reductions in perceived stress and improvements in stress-related biomarkers among healthy adults.

The mechanisms underlying Tai Chi's stress-reducing effects likely involve multiple pathways. Research suggests that Tai Chi practice activates the parasympathetic nervous system, reducing cortisol levels and promoting relaxation responses (31). Additionally, the meditative components of Tai Chi may enhance emotion regulation capacity through improved prefrontal cortex function and reduced amygdala reactivity (5). The mindful attention to movement and breath characteristic of Tai Chi practice may facilitate adaptive coping strategies and reduce maladaptive stress responses (32). It should be noted that the physiological and neurobiological mechanisms discussed above were not directly measured in the present study. Therefore, these explanations should be interpreted as theoretical mechanisms derived from previous literature rather than direct evidence from the current investigation. Future research incorporating physiological markers such as autonomic measures, cortisol levels, or neuroimaging techniques would help clarify the underlying mechanisms of Tai Chi's psychological benefits.

**Figure 2:**
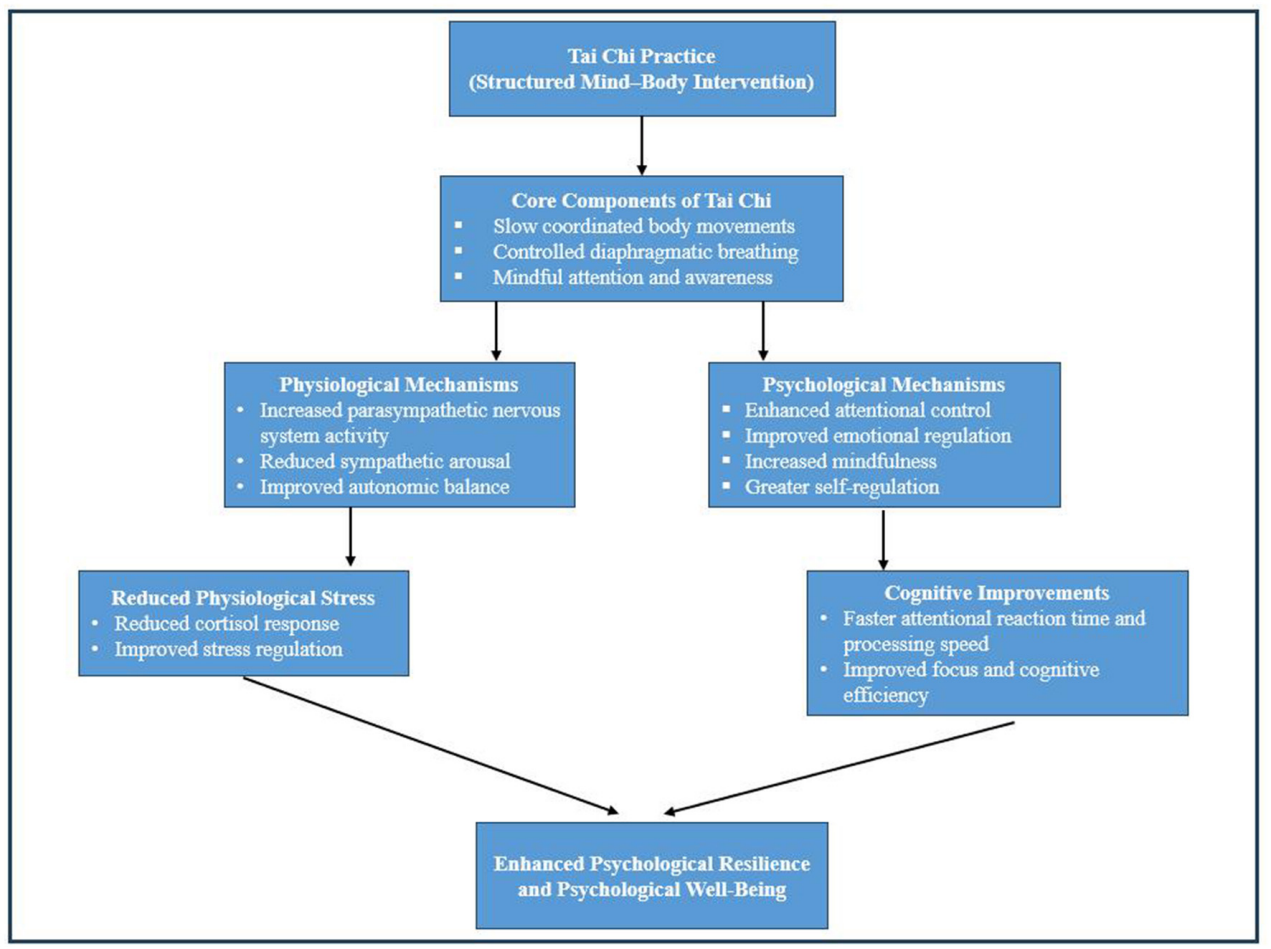
Conceptual framework illustrating the potential mechanisms underlying the psychological benefits of Tai Chi practice.

The potential physiological and psychological mechanisms through which Tai Chi practice may influence stress regulation, attentional performance, and psychological resilience are illustrated in Figure 2.

Tai Chi integrates slow coordinated body movements, controlled breathing, and mindful attention. These elements may influence both physiological and psychological pathways. Physiologically, Tai Chi practice may enhance autonomic nervous system regulation by increasing parasympathetic activity and reducing sympathetic arousal, thereby improving stress regulation. Psychologically, the attentional and mindfulness demands of Tai Chi practice may strengthen cognitive control and emotional regulation processes. Together, these mechanisms may contribute to reduced perceived stress, improved attentional performance, and enhanced psychological resilience.

In addition to these explanations, Tai Chi practice may influence psychological outcomes through integrated physiological and cognitive pathways. The coordinated interaction between slow body movements, diaphragmatic breathing, and mindful attention may enhance parasympathetic nervous system activity while reducing sympathetic arousal. This autonomic regulation may improve stress management and emotional balance. Simultaneously, the requirement to sustain attention during movement sequences may strengthen attentional control networks within the brain, thereby enhancing cognitive efficiency. Over time, improvements in stress regulation and attentional control may contribute to greater psychological resilience by strengthening individuals' capacity to adapt to stress and environmental challenges.

The moderate-to-large effect size observed for stress regulation (η^2^p = 0.32) in the present study suggests that Tai Chi may be particularly effective as a stress management intervention. This finding has important implications for public health, as chronic stress represents a significant risk factor for numerous physical and mental health conditions, including cardiovascular disease, depression, and immune dysfunction (33). The accessibility and low cost of Tai Chi make it an attractive option for population-level stress reduction interventions.

Participants in the Tai Chi group demonstrated significant improvements in attentional performance, evidenced by a reduction in reaction time of 47.76 milliseconds (*p* = 0.001), while the control group showed no significant change. Accuracy scores on the Attention Network Test did not show statistically significant changes between pre- and post-intervention assessments. These results corroborate previous findings indicating that Tai Chi practice enhances cognitive function, particularly attentional control. A meta-analysis by Wayne et al. (7) reported that Tai Chi interventions were associated with improvements in executive function, including attentional processes, across both healthy populations and clinical samples.

The attentional demands inherent in Tai Chi practice may contribute to these cognitive benefits. Tai Chi requires sustained attention to bodily sensations, movement coordination, and environmental awareness, thereby providing regular training in attentional control (12). Neuroimaging studies have demonstrated that mind-body practices like Tai Chi are associated with structural and functional changes in brain regions involved in attention, including the anterior cingulate cortex and dorsolateral prefrontal cortex (34).

Furthermore, research by Chang et al. (35) found that 12 weeks of Tai Chi practice improved performance on tasks measuring selective attention and response inhibition in middle-aged and older adults. The present study extends these findings by demonstrating attentional improvements in a broader adult sample using objective reaction time measures. The moderate effect size observed for attention (η^2^p = 0.16) suggests that Tai Chi represents a viable intervention for enhancing cognitive performance in the general population. These findings are particularly relevant given the increasing demands on attentional resources in modern society and the documented associations between attentional deficits and reduced quality of life, academic and occupational performance, and increased accident risk (36). The accessibility of Tai Chi makes it a promising intervention for addressing attention-related challenges at the population level.

The most substantial effect observed in the present study was on psychological resilience, with the Tai Chi group showing a significant increase of 9.50 points (*p* < 0.001, η^2^p = 0.41), representing a large effect size. The control group exhibited no significant change. These findings align with emerging evidence suggesting that mind-body interventions can enhance psychological resilience, the capacity to adapt successfully to stress and adversity (4).

A randomized controlled trial by Caldwell et al. (37) demonstrated that Tai Chi Chuan practice significantly improved psychological resilience among college students experiencing high levels of academic stress. Similarly, research by Abbott and Lavretsky (38) found that Tai Chi enhanced resilience and reduced depressive symptoms in older adults. The present study extends these findings by demonstrating resilience-building effects in a general adult population using a standardized resilience measure.

The mechanisms through which Tai Chi enhances resilience may be multifaceted. Tai Chi practice cultivates mindfulness, self-efficacy, and interoceptive awareness, all factors associated with greater psychological resilience (39). Additionally, the social support inherent in group-based Tai Chi practice may contribute to resilience through enhanced social connectedness and reduced isolation (40). The mind-body integration fostered by Tai Chi may also promote more adaptive responses to stressors by enhancing the capacity for self-regulation and emotional balance (41).

The large effect size observed for psychological resilience in the present study has important implications for mental health promotion and prevention. Given that resilience serves as a protective factor against the development of psychiatric disorders and promotes recovery from adverse life events (42), interventions that enhance resilience have the potential to reduce the burden of mental illness at the population level. The scalability and cultural adaptability of Tai Chi make it particularly well-suited for implementation as a public health resilience-building intervention.

The convergent improvements observed across stress regulation, attention, and psychological resilience suggest that Tai Chi exerts broad beneficial effects on psychological functioning. These findings are consistent with the conceptualization of Tai Chi as a holistic mind-body practice that integrates physical movement, mental focus, and emotional regulation (43). The interconnected nature of these outcomes is supported by theoretical models suggesting that improvements in one domain (e.g., stress regulation) may facilitate gains in others (e.g., attention and resilience) through common underlying mechanisms such as enhanced self-regulation capacity (44).

From a public health perspective, the present findings suggest that Tai Chi represents a promising scalable intervention for promoting psychological wellbeing across the adult population. Several characteristics make Tai Chi particularly well-suited for public health implementation. First, Tai Chi is low-cost and requires minimal equipment, making it accessible to diverse socioeconomic groups (45). Second, Tai Chi can be practiced across the lifespan and adapted to various fitness levels and physical abilities (46). Third, Tai Chi's cultural acceptability and enjoyment may promote sustained engagement and adherence, addressing a common challenge in public health interventions (47).

The present study has several strengths, including the use of a randomized controlled design, standardized outcome measures, and rigorous statistical analyses that confirmed the normality of data and accounted for multiple comparisons. The significant Group × Time interactions with large effect sizes provide strong evidence for the efficacy of the Tai Chi intervention.

However, several limitations should be acknowledged. First, the study did not include long-term follow-up assessments, limiting conclusions about the durability of intervention effects. Future research should examine whether the observed improvements are maintained over extended periods and identify factors that promote sustained practice. Second, the mechanisms underlying the observed effects were not directly assessed. Future studies incorporating biomarkers, neuroimaging, or process measures (e.g., mindfulness, self-efficacy) would provide valuable insights into how Tai Chi produces psychological benefits. Third, while the present study demonstrated efficacy in a general adult sample, the extent to which findings generalize to specific populations (e.g., individuals with clinical levels of stress or psychiatric disorders) remains to be established. Fourth, the study did not control for potential confounding variables such as social interaction or exercise expectancy effects, which may have contributed to the observed outcomes. Future research employing active control conditions (e.g., stretching or social activities) would help isolate the specific effects of Tai Chi practice. Finally, adherence and practice frequency were not systematically assessed beyond attendance records. Future studies should examine dose-response relationships between Tai Chi practice intensity and psychological outcomes to inform optimal implementation strategies.

Another limitation relates to the multi-component structure of the intervention sessions, which included warm-up exercises and relaxation or breathing practices in addition to Tai Chi movements. Although these elements are typically integrated within traditional Tai Chi training sessions, it is possible that the observed psychological benefits may partly reflect the combined influence of gentle physical activity, breathing regulation, and relaxation processes rather than Tai Chi movements alone. Therefore, caution should be exercised when attributing the observed effects exclusively to Tai Chi practice. Future studies employing active control conditions or isolating specific intervention components would help clarify the relative contribution of Tai Chi to the observed improvements. Another important limitation concerns the use of a passive control group that continued routine daily activities without receiving a structured intervention. As a result, factors such as social interaction, group engagement, and expectancy effects were not controlled in the present study. These factors may partially contribute to the improvements observed in the intervention group. Future studies should consider the use of active control conditions, such as stretching programs, walking interventions, or health education sessions, to better isolate the specific effects of Tai Chi practice and strengthen causal interpretation.

## 5 Conclusions

This study provides robust evidence that Tai Chi significantly improves stress regulation, attention, and psychological resilience in adults, with moderate to large effect sizes across all outcomes. The intervention group demonstrated substantial reductions in stress levels, notable improvements in attentional performance, and marked increases in psychological resilience compared to controls. These findings support Tai Chi as an evidence-based mind-body intervention with multidimensional psychological benefits. From a public health perspective, Tai Chi's accessibility, low cost, and adaptability across diverse populations position it as a scalable intervention for promoting mental health at the population level. Future research should examine long-term sustainability of effects, underlying mechanisms, and cost-effectiveness to further establish Tai Chi's role in comprehensive public health strategies for enhancing psychological wellbeing and preventing stress-related disorders.

## Data Availability

The original contributions presented in the study are included in the article/Supplementary material, further inquiries can be directed to the corresponding author.
