# Supplementary material for: Tai Chi as a public health intervention: effects on stress regulation, attention, and psychological resilience in adults
Source: Front Public Health. 2026 May 4;14:1791759. doi: 10.3389/fpubh.2026.1791759 (PMC13180939; doi:10.3389/fpubh.2026.1791759)
Supplement: Supplementary file 2 [file Data_Sheet_1.pdf]

frontiers

Supplementary Material

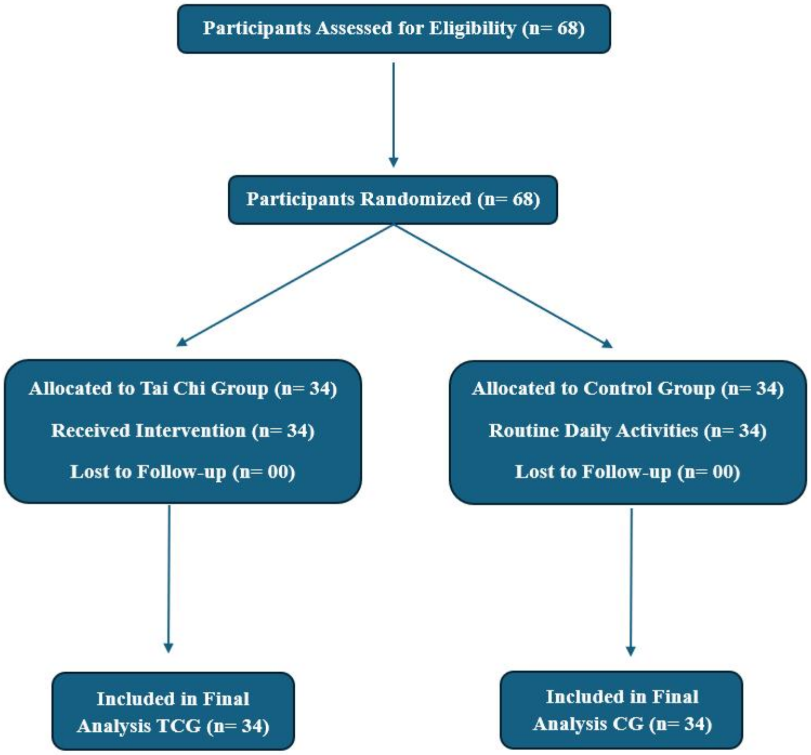

Supplementary Figure 1. CONSORT flow diagram of participant recruitment, randomization, allocation, follow-up, and analysis This figure illustrates the flow of participants through the randomized controlled trial. A total of 68 adults were assessed for eligibility and randomly allocated to either the Tai Chi intervention group (TCG, n = 34) or the control group (CG, n = 34). Participants in the TCG received an 8-week structured Tai Chi training program, while participants in the CG continued with routine daily activities. No participants were lost to follow-up, and all randomized participants were included in the final analysis.
